# Systematic Exposition of Mesenchymal Stem Cell for Inflammatory Bowel Disease and Its Associated Colorectal Cancer

**DOI:** 10.1155/2018/9652817

**Published:** 2018-12-26

**Authors:** Jingjing Kang, Li Zhang, Xiao Luo, Xiangyu Ma, Gaoying Wang, Yanhui Yang, Yongmin Yan, Hui Qian, Xu Zhang, Wenrong Xu, Fei Mao

**Affiliations:** ^1^Key Laboratory of Medical Science and Laboratory Medicine of Jiangsu Province, School of Medicine, Jiangsu University, Zhenjiang, Jiangsu 212013, China; ^2^Nanjing Lishui People's Hospital, Zhongda Hospital Lishui Branch, Southeast University, Nanjing, Jiangsu 211200, China; ^3^The Third People's Hospital of Sihong County, Suqian, Jiangsu 223911, China

## Abstract

Mesenchymal stem cells (MSCs) therapy has been applied to a wide range of diseases with excessive immune response, including inflammatory bowel disease (IBD), owing to its powerful immunosuppression and its ability to repair tissue lesions. Different sources of MSCs show different therapeutic properties. Engineering managements are able to enhance the immunomodulation function and the survival of MSCs involved in IBD. The therapeutic mechanism of MSCs in IBD mainly focuses on cell-to-cell contact and paracrine actions. One of the promising therapeutic options for IBD can focus on exosomes of MSCs. MSCs hold promise for the treatment of IBD-associated colorectal cancer because of their tumor-homing function and chronic inflammation inhibition. Encouraging results have been obtained from clinical trials in IBD and potential challenges caused by MSCs therapy are getting solved. This review can assist investigators better to understand the research progress for enhancing the efficacy of MSCs therapy involved in IBD and CAC.

## 1. Introduction

Inflammatory bowel disease (IBD) is a type of intestinal mucosal inflammation in the colon and small intestine. IBD typically includes Crohn's disease (CD) and ulcerative colitis (UC). The pathogenesis of IBD is complex; however, scholars have indicated that hereditary and environmental factors elicit intestinal immune system disorders and mucosal damage. Furthermore, the protracted course of colitis can easily trigger chronic enteritis and eventually induce colon cancer such as colitis-associated cancer (CAC) stimulated by external oncogenic factors [1, 2]. Patients with chronic colitis exhibited a 2- to 8-fold risk of carcinogenesis compared with others without [3].

Traditional therapy for IBD mainly consists of surgery [4] and medicine therapies [5, 6]; the former is invasive and of high risk and the latter can not treat the underlying danger. The clinical remission rates of these therapeutic methods for IBD are 20%–30%, but remission could reach approximately 50% by using combinations of therapies [7]. Effective treatment options were seldom achieved in colitis-associated CRC (CAC). The majority of patients underwent cancer lesion removal through surgical resection, and this treatment was typically supplemented by chemotherapy and radiotherapy [8].

MSCs therapy is a novel strategy for IBD [9] and CAC [10] owing to easily detachable characteristics, low immunogenicity, and the favorable environment for tissue regeneration compared with traditional therapy [11]; MSCs were utilised in the treatment of IBD and CAC [12] with the relevant investigation techniques developed and challenges surmounted. MSCs do not generally exert strong immunogenicity in immune-dominated diseases because of the difficulty caused by HLA and obtain strong immunosuppression in IBD and CAC [13]. In light of a wide variety of studies, this review aims to investigate the recent research advances of MSCs therapy for IBD and IBD-associated CRC.

## 2. Pathogenic Mechanism Involved in IBD

**Figure 1:**
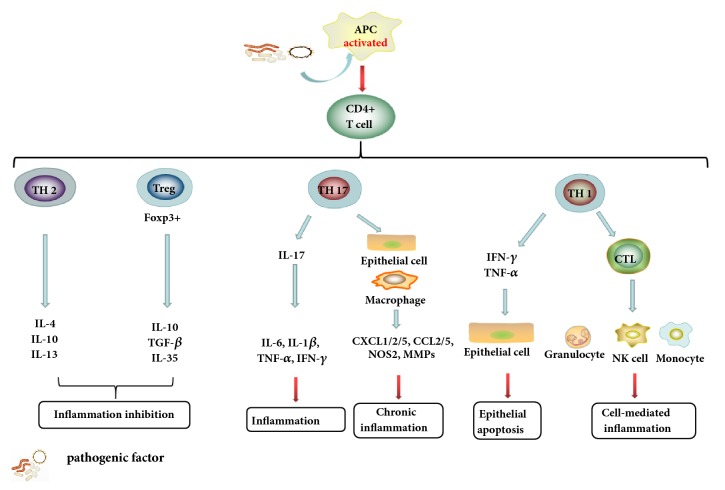
**The major active procedure of adaptive immune response involved in IBD.** CD4+T cells diverted into diverse phenotype under the stimulus of the pathogenic factors and then secreted proinflammatory or anti-inflammatory to exert different disease effects.

Aetiological agent of IBD is complex and unknown, either UC or CD; the most fundamental pathogenesis pattern involved in IBD is the excessive activation of innate and adaptive immune responses, the former being the first line of defense against pathogenic factors and the latter being considered as the main driver of disease occurrence [14]. CD4+ T cells activated by pathogenic factor can differentiate into CD4+ T-helper (Th) cells which mainly refer to CD4+ Th1 cells and CD4+ Th17 cells and promote the production of proinflammatory M1 macrophages or other immune cells. Both of CD4+ Th1 cells and CD4+ Th17 cells can release a variety of inflammatory cytokines to trigger intestinal epithelial inflammatory cells infiltrate and acute or chronic enteritis. However, intestinal epithelial inflammation would be suppressed via the differentiation of CD4+FoxP3+T regulatory cells (Tregs) and the supplementary of CD4+ Th2 cells. IL-10 and TGF-*β* secreted from Tregs create a kind of immunosuppressive microenvironment to facilitate the repair of gastrointestinal tract dysfunction and the colon mucosal lesion [15] (Figure 1).

## 3. Different Sources of Mesenchymal Stem Cells Involved in IBD

BMMSCs therapy was the most widely employed allogeneic-based stem cells therapy in laboratory investigations or clinical science studies [16]. BMMSCs infusion facilitated intestinal mucosal permeability reconstruction and oxidative stress relief and exerted neuroprotective function in 2,4,6-trinitrobenzene sulfonic acid colitis, which depended on the number of doses [17, 18]. BMMSCs also can exert long-term protective effects on dextran sulfate sodium salt (DSS)-induced chronic colitis [19]. Nevertheless, the acquisition of BMMSCs is invasive and painful; therefore these inadequacies tremendously restrict the application of BMMSCs in clinical regenerative medicine [20].

AD-MSCs offered similar immunosuppressive function but more convenient methods of obtaining materials than BMMSCs [21]. A study reported that the quantity of MSCs cultured from each gram of the adipose tissue is higher than that derived from a gram of the myeloid tissue [22]. AD-MSCs mainly originated from the epiploon adipose tissue in rats [23], the epididymis and inguinal fat originated from mice [24], and the processed lipoaspirate was drawn from human bodies [25]. AD-MSCs can migrate to lymph nodes to contribute to immunomodulatory responses and exert more therapeutic effects on CD that are similar to those of BM-MSCs [25, 26]. Both types of AD-MSCs and BM-MSCs could reach the intestinal muscularis to result in colitis remission; compared with AD-MSCs, BMMSCs offered more advantages in enteric neuropathy and plexitis [27]. Hence, although AD-MSCs are effective, BMMSCs should be used for some diseases during the exceptional circumstances.

UCMSCs are also a promising therapeutic materials for experimental and clinical IBD owing to their noninvasive and painless sampling feature [28, 29]. UCMSCs contain umbilical cord blood (UCB) and the interstitium surrounding the umbilical cord vein [30]. Similar to AD-MSCs, UCMSCs also exerted stem cell characteristics in relation to BMMSCs [31]. Investigators performed a sequence of experiments to compare the proliferative and differentiation capacities between BMMSCs and UCMSCs; the results showed that UCMSCs were preferable to BMMSCs from comprehensive respect [32]. Mao et al. reported that HUCMSCs could alleviate IBD which is mainly through the downregulation of the 15-lox-1 expressed in the macrophage and through the reduction of STAT3 phosphorylation [33].

Other sources of MSCs have been applied in IBD and CAC which mainly include tissues from genitals and the oral cavity. Stem cells from genitals include endometrial regenerative cells (ERCs) [34], amniotic fluid stem cells (AFSCs) [35], placenta-derived MSCs (PMSCs) [36], and amnion-derived MSCs (AMSs) [37]. AFSCs grow from the second-trimester amniotic fluid of humans and could unite into the intestinal mucosa to reduce intestinal epithelial dysfunction [35, 38]. From 1988 to 2018, studies have gradually reported that ERCs derived from menstrual blood were able to regulate series of immunological responses between CD3+CD25+ T lymphocytes and CD4+CD25+Foxp3+ Treg cells in IBD [34]. MSCs from the oral cavity environment contain gingiva-derived MSCs (GMSCs) [39] and tonsil-derived MSCs (TMSCs) [40]. GMSCs were mainly developed from the cranial neural crest and promoted immune tolerance and autophagy in IBD [39] and TMSCs and could exert therapeutic effects on IBD [40]. Different MSCs can secrete different levels of soluble factors, thus exerting diverse suppressive actions on antigen presenting cells (APCs), and unequal therapeutic sensitivities to different diseases because of their heterogeneity. Therefore, which type of MSCs should be choosed depending on the circumstances [41, 42].

## 4. Engineered Mesenchymal Stem Cells Involved in IBD

In Phase II and Phase III clinical trials of MSCs for diseases, including IBD and CAC, various problems have arisen and urged researchers to develop sound strategies [43]. The proinflammatory or anti-inflammatory features of MSCs might produce completely opposite manifestations under inflammatory environments [44]. The most cumbersome challenge of MSCs in clinical IBD and CAC therapy was their low efficiency which means low survival rate and immunosuppressive capacity in the process of homing to the intestinal mucosa [45]. For this reason, multiple valid MSCs engineering managements have been applied for IBD and CAC to solve these difficulties; these pretreatments mainly can be divided into two types: those that enhance the immunomodulation function and those that improve survival both* in vitro* and* in vivo*.

### 4.1. To Enhance Immunomodulation Function of MSCs Involved in IBD

The immunomodulation function of MSCs in IBD would be boosted by using several methods, including the genetic modification of MSCs, realized through overexpression induced by the transfection of a plasmid, adenovirus, and lentivirus; through downregulation by siRNA [46]; and through external chemical reagents. MSCs combined with microRNA (cell function modulators), a kind of 24-bp noncoding RNAs, have been applied in safe treatment strategies [47]. Some BMMSCs derived from miR-21^−/−^ mice expressed additional transforming growth factor (TGF)-1 to promote immunosuppression [48]. IFN-*γ* transfection and IGFBP7 knockdown both could transform CD4+ and CD8+ cytokines generation and boosted MSCs proliferation [49]. Moreover, increasing the expression of CXCR4 could accelerate the homing of BMMSCs to intestinal injury lesions [50]. Although gene modification offered various advantages, it entails some carcinogenic hazards [45].

External chemical reagents also can boost the efficiency of MSCs migration to the bowel mucosa to improve immunoregulation [51, 52]. MSCs pretreament with TLR3 could secrete extra immunosuppressive cytokines and inhibit active T cells proliferation; TLR4 aggravated the process of enteritis [53]. BMMSCs, treated with acetylsalicylic acid (ASA), released additional monocyte chemotactic protein-1 (MCP-1) to recruit T cells to cause apoptosis through the 15d-PGJ2/PPARg/TGF-b1 pathway [45]. However, strategies for enhancing immune function in MSCs did not show any beneficial effects in the preclinical trials of IBD treatment, because the immature technology caused a series of serious immunoreactions in practice [54].

### 4.2. To Improve Survival Both In Vitro and In Vivo of MSCs Involved in IBD

Improvement of the survival and multipotency of MSCs could be realized with the melatonin, along with three other ingredients, namely, rapamycin, resveratrol, and quercetin, to fight senility and to form additional colonies* in vitro* [55]; MSCs also could be conserved and moved more easily in spheroidal shape to play a highly effectual role in the treatment of experimental enteritis* in vivo* [56, 57]. Surprisingly, a team in China used heparin to reduce coagulation factor during the transport of MSCs in the blood vessel to obtain more security in inflammatory colitis, which solve the high risks of pulmonary embolism, cardiac failure, and mortality [58]. In order to better understand the current research progress of MSCs with engineering arrangements in IBD, the therapeutic effects of MSCs from different sources with pretreatment are listed in Table 1.

## 5. Therapeutic Theories of MSCs Involved in IBD

**Figure 2:**
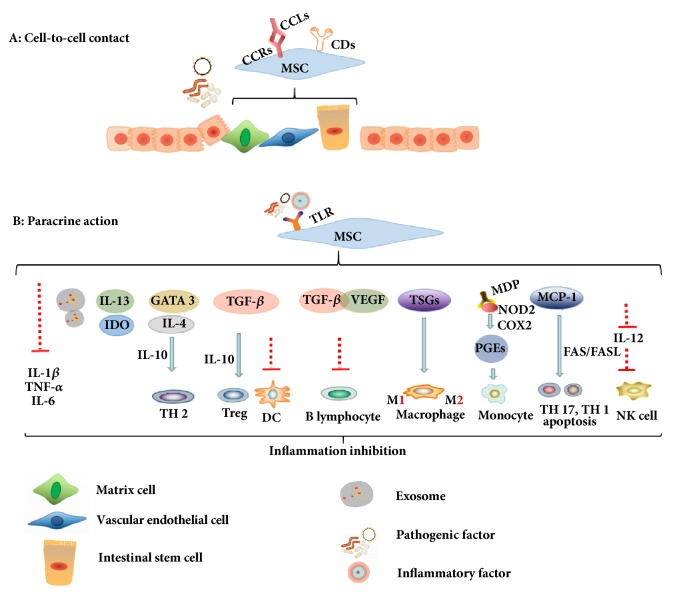
**MSCs in IBD and CAC mainly aim to suppress overactive immune responses by cell-to-cell contact and paracrine action.** A: MSCs can integrate into intestinal mucosa by the differentiation of intestinal epithelial cells, then format vascular endothelial cells and matrix cells to restore intestinal wall parclose. B: The immunosuppressive effect of MSCs on IBD is mainly owing to paracrine function which secreted different cytokines to inhibit inflammation. MSCs also can secrete exosomes to the intestinal mucosa to inhibit inflammation.

The therapeutic theories of different sources of MSCs were similar but also put different emphasis in IBD, compared in Table 2, and were mainly aiming to suppress overactive immune responses by anti-inflammatory and immunosuppressive effects. Briefly, it can be summarized into the combination of cell-to-cell contact and paracrine actions aiming at each pathogenic link point subtly and validly as deduced in Figure 2 [71, 72].

### 5.1. Cell-to-Cell Contact

Different MSCs administration all can be effective to migrate to the designated area of intestinal injury when IBD happened [73]. The homing functions were owing to the fact that MSCs expressed many chemotaxis receptors such as CCR1, CCR2, CCR4, CCR5, CCR9, CXCR1, and CXCR5; the CCR2 and CCR4 were major migration helpers of MSCs in colitis, and CXCR4 combined with SDF-1 can promote MSCs migration [74]. Those receptors in MSCs mainly bind to their specific ligands which mainly include CCL5, CCL19, CCL22, CCL25, CXCL8, and CXCL13 that are released by some kinds of histiocyte in enteritis environment [75]. Some adhesion molecules expressed in the membrane surface of MSCs also took part in IBD remission containing CD29, CD44, CD49e, CD54, CD105, CD106, and CD166 [74]. MSCs were able to differentiate and integrate into intestinal epithelial cells and then restore vascular endothelial cells and matrix cells, but the proficiency of tissue regeneration capacity could be destroyed due to the serious enteritis [76, 77]. However, the strong immunosuppressive effect on IBD was largely owing to paracrine function because of the poor transitory persistence and survival ability of MSCs [78].

### 5.2. Paracrine Actions

The paracrine functions of MSCs are much complex and controversial in IBD but can be roughly into a simple logic. Pathogenic factors and inflammatory mediators integrate with different types of toll-like receptor or other manners to initiate the process of excreting anti-inflammatory cytokines of MSCs such as indoleamine 2,3-dioxygenase (IDO) [68], transforming growth factor beta (TGF-*β*), IL-4, IL-10, IL-13, GATA3, and prostaglandins (PGEs) [79], among others. Those immunosuppressive agents can inhibit granulocyte infiltration such as T cells, B cells, NK cells, and antigen presenting cells (APCs), even dendritic cells (DCs), to exert immunosuppressive function through a variety of channels.

MSCs provided vascular endothelial growth factor receptor (VEGF) and TGF-*β* to promote angiogenesis and tissues repair in colon region [80] and restrained the proliferation of B lymphocyte via promoting the expression of CD40 in colitis [81]. MSCs retarded the production of IL-12 to control the function of NK cells and the secretion of TGF-*β* to inhibit the maturity, activation, and antigen presentation of dendritic cells [72]. Thus, nucleotide-binding oligomerization domain 2 (NOD2) signal channel would be activated by MSCs to boost the cyclooxygenase-2 (COX-2) and PGE2 expression and reduce the multiplication of monocyte to ease enteritis [31]. MSCs also could restrain macrophages activation and secrete tumor-specific glycoproteins (TSGs) to transform the phenotype of macrophages from M1, identified as proinflammatory properties, to M2, which is recognized as anti-inflammatory maker directly by secretion of soluble cytokines such as TGF-*β* in UC and CD [82]. Meanwhile, MSCs were able to excrete monocyte chemotactic protein-1 (MCP-1) to repress the activation and promote the apoptosis of CD4+ T cells via the FAS/FASL impact, thus inhibiting T lymphocyte proliferation in IBD [83, 84]. Some scholars summarized that MSCs could lessen the infiltration of Mac-1 positive cells in the colon tract and DC cells in the spleen and generate TGF-*β*, IL-10, and Foxp3+ factor to promote CD4+CD25+Foxp3+ regulatory T cells activation and secrete IL-4, IL-10, and GATA3 to advance T helper cells 2 (Th2) creation to execute inflammation resistance. In the meantime, the reduction of IL-2, TNF-*α*, IFN-*γ*, T-bet, IL-6, and IL-17 could induce both the Th1 and Th17 cells proliferation regarded as inflammatory leader in IBD [34, 79]. Some researchers detected that exosomes derived from different sources of MSCs, which have been recognized as a kind of paracrine interaction, also exerted strong immunosuppressive effects in IBD. We will explain its features and functions below.

### 5.3. Signaling Pathways

**Figure 3:**
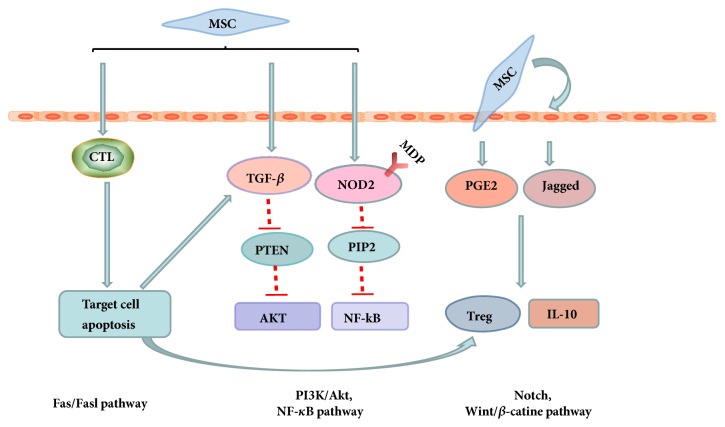
**Signaling pathways involved in the IBD therapy of MSCs.** Signaling pathways are not independent but relevant and cooperate with each other closely in IBD.

The signaling pathways involved in the IBD of MSCs therapy are mainly in the aspect of the immunology and inflammation (NF-*κ*B pathway) [37, 85], the developmental biology signaling (wnt/*β*-catenin signaling interactive pathway and Notch signaling interactive pathway) [45, 69], PTEN/PI3K/Akt signaling resources [48], and apoptosis resources (FAS/FASL) [65]. Those signaling pathways are not independent but relevant and cooperate with each other closely (Figure 3).

BMMSCs derived from miR-21^−/−^ mice inhibited the gene of phosphate and tension homology deleted on chromosome ten (PTEN), due to the secretion of TGF-*β*1, and consequently suppressed the AKT activation which was the downstream of PTEN [48]. NOD2 binding to its ligand, named as MDP expressed in HUCMSCs, may inhibit the activation of RIP2, a kind of caspase like apoptosis regulatory protein kinase, and then descend the expression of NF-*κ*B in the CD experiment model similarly [31]. Notch signaling interactive pathway activated by MSCs is a kind of cell to cell interaction which has no second messenger and only is able to affect adjacent cells, promote the ascend of PGE2 and Jagged-1 production, and then increase the secretion of IL-10 and the proliferation of Treg cells in colitis [69, 80]. Wint/*β*-catenin signaling interactive pathway plays the curative role through participating in the MSCs adhesion procedure in colitis [86]. Fas/Fasl pathway, a kind of membrane surface molecules related to cell apoptosis, mediates its targeting cells apoptosis induced by cytotoxicity in T cell development and upregulates TGF-*β* which facilitated the Treg cells expansion and promoted the immunologic tolerance in colitis [45, 87].

To sum up, mechanisms of MSCs therapy involved in colitis are complicated and multiple and mainly rely on the cooperation of cell-to-cell and paracrine actions accompanied with NF-*κ*B, Wnt/*β*-catenin, PI3K/Akt, and FAS/FASL signaling pathway and so on.

## 6. Exosomes

Exosomes are a kind of membranous vesicles, produced by endocytosis, containing a variety of proteins, nucleic acid, and other active substances [88, 89]. Exosomes also are the medium of intercellular material transport and signal transduction, which are more penetrating and secure compared with MSCs in IBD [90, 91]. It was detected that the number of MSCs administered within the safe range of quantity may not be sufficient to protect the injured intestinal tissues, which means that the additional mechanisms exist. Exosomes derived MSCs were important to remit acute tubular injury [92] and myocardial ischemia/reperfusion damage [93], indicating that exosomes have the similar therapeutic ability to be applied in inflammatory diseases. Actually, it is inspiring that Mao et al. formulated that exosomes derived from HUCMSCs inhibited the expression of iNOS and IL-7 during coculture with mouse enterocoelia macrophages [94]. It was also reported that extracellular vesicles derived from BMMSCs (BMSC-EVs) could rebuilt the intestinal barrier [95]. Some researchers have made a comparative discovery that BMMSCs derived exosomes obtained independently repair function of intestinal nerve compared with BMMSCs. However, this feature was no longer available with the culture supernatant of MSCs removing the exosomes cocultured with scrape wounding IEC* in vitro* [96].

**Figure 4:**
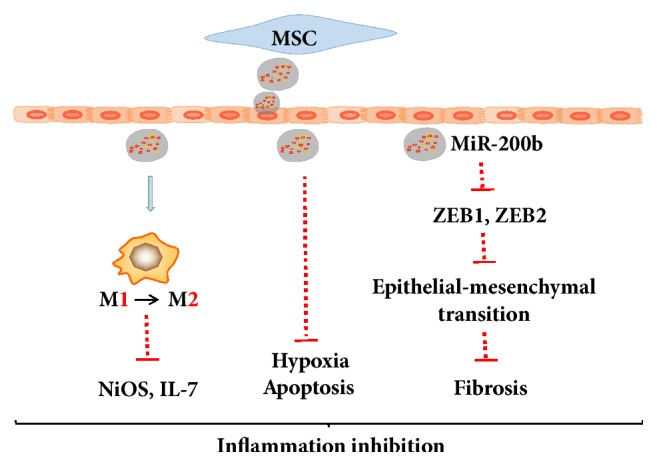
**Exosomes are the medium of intercellular material**. transport and signal transduction.

The contents of the exosomes are specific to the origin of cells and BMMSCs derived exosomes have be proved possessing over 730 proteins, many of which promote self-renewal and differentiation [97]. Recently, studies have also shown that BMMSCs-derived exosomes contain more than 300 miRNAs, which are noncoding small RNAs that direct the silencing complex (RISC) to degrade mRNA or hinder its translation by base pairing with the target gene mRNA [98]. Although it has not been reported which miRNA in the exosomes of MSCs may effectively participate in and regulate the repair of enteritis, some researchers have summarized that miR-21, miR-146a, and miR-181 derived from exosomes of HUCMSCs were able to effectively regulate wound healing [99]. We described a concise schematic according to the present literature in Figure 4. Compared to MSCs, exosomes derived from MSCs obtain more security whereas the MSCs could bring out the risk of death because of coagulation factor and so on [100]. Therefore, the application of exosomes will be a promising therapeutic option to inflammatory bowel disease.

## 7. MSCs Therapy in Colorectal Cancer

MSCs therapy in colorectal cancer was controversial and debatable because the powerful immunosuppression function of MSCs resisted the therapy effects on the colorectal cancer, including hereditary, sporadic, and colitis-associated colorectal cancer (CAC), which mainly resulted from immune evasion in the tumor microenvironment [101]. However, MSCs still exist a potency to ameliorate the colorectal cancer under certain circumstances [102].

### 7.1. For Metastatic Tumor and Complications from the Colorectal Cancer

MSCs may be applied to the metastatic tumor and complications resulted from the colorectal cancer. MSCs derived from human term placenta expressing endostatin could reduce the number of peritoneal carcinomatosis node and prolong the survival [36]. MSCs can also serve as gene delivery vehicles targeting the solid neoplasms tissue. It was reported that MSCs overexpressed oncolytic virus inhibited the ovarian cancer development strongly and MSCs embellished with IL-12 was able to slow cancer growth [103]. Similarly, MSCs processed to excrete pigment epithelium derived factor (PEDF), which reduced the angiogenesis via lessening the expression of VEGF, could depressed tumor metastasis and the formation of malignant ascites [104]. This feature may be owing to the targeting neoplasm and immune inhibitory capacity of MSCs, which ensured MSCs would not be dissolved by cytotoxic lymphocyte compared with other active factor utilized in colorectal cancer [104, 105]. Moreover, the tumor microenvironment also facilitated the homing migration and survival of MSCs [106]. Therefore, MSCs are the favorable promoter and support to transport therapeutic genes in CAC.

### 7.2. For Colitis-Associated Colorectal Cancer (CAC)

MSCs obtained a powerful therapeutic function in colitis-associated colorectal cancer (CAC), which deteriorated from chronic enteritis and tends to occur in the region of chronic inflammation [3]. HUCMSCs migrated to the intestinal structure and then lengthened the colon and reduced the number of tumors with the reduction of Ki67 via inhibiting chronic inflammation and Smad2 signaling pathway [10]. However, Nasuno et al. considered that MSCs just reduced the number of tumors but not the size. In other words, MSCs played a role of chemoprophylaxis to eliminate the occurrence of tumor depending on the Wnt/*β*-catenin pathway and TGF-*β*-Smad pathway with influencing the cell cycle remaining in the G1 phase and promoting the apoptosis of enteric tumor cells [107]. There were also some other scholars who discussed that BMMSCs could remiss the CAC via reducing the STAT3 phosphorylation company with the retard of weight loss and decrease of proinflammatory factors expression [108]. In vitro, BMMSCs secreting specific cytokines accelerated the devastation and depressed the proliferation of colonic cancer cells via inhibiting the PI3K/AKT pathway and extracellular signal-regulated protein kinase (ERK) expression in the administration of low doses of UV irradiation or X-rays [109].

In the tumor microenvironment, MSCs regulated the immunologic function by altering the cytokines secreted from APC cells, NK cells, and T cells and this property owned dual functions because MSCs could promote both the apoptosis and survival of tumor cells [110]. Therefore, the application of MSCs will be a promising strategy for colorectal cancer but the techniques and procedures still need to be considered twice especially that the therapy effects of MSCs are controversial and only occur under certain conditions of CAC.

## 8. Clinical Progress and Challenges of MSCs Involved in IBD

MSCs therapy in IBD mainly aimed to repair inflammation, reduced the number of surgeries, improved the quality of life of patients, and thus cut down the cost of living. As is mentioned above, MSCs whether derived from different sources or engineered with different agents both obtained strong immunosuppression in preclinical stage. Meanwhile, the clinical research of MSCs involved in IBD also produced a rich harvest and a number of experts have demonstrated the positive effects of MSCs on ulcerative colitis and Crohn's disease from 1993 to the present [111]. But the challenges were growing with the development of MSCs therapy in IBD including which source of MSCs to apply, when, how, and in what doses and what the side effects are. We summarized the clinical data of representative MSCs therapy for the treatment of enteritis from* Pubmed *in the past decade (2008~2018) and listed some common features and characteristics (Table 3).

### 8.1. Which Source of MSCs to Choose?

BMMSCs are the most used in IBD and researchers found that three out of ten patients are effective to the MSCs therapy [117]. However, BMMSCs are invasive and have fewer donors compared with other sources of MSCs; thus the application of ADMSCs is becoming more and more. Similar to animal experiments, there was no significant difference between ADMSCs and BMMSCs in clinical treatment and ADMSCs were convenient for* in vitro* expansion, which inhibited the proliferation of activated lymphocytes to alleviate systemic and local inflammation [122]. UCMSCs also could ameliorate endoscopic indices and suppress lymphoid infiltration via exerting immunosuppressive effect in patients with serious IBD [123]. The objective evaluation of differences in clinical efficacy of MSCs from different sources is still ambiguous because of the limited number of clinical trials.

Both autogenous and allogeneic sources of MSCs have been demonstrated as effective for the treatment of enteritis. It was sufficient for autogenous MSCs to expand* in vitro* during the long course of IBD and researchers reported that BMMSCs isolated from patients themselves revealed similar immunosuppression function and biological characteristics compared with allogeneic MSCs from healthy donors [117, 124]. However, whether certain genetic defects or insufficient immunosuppressive functions exist in their autologous MSCs for patients with severe IBD was still unknown [125]. Allogeneic MSCs may circumvent these deficiencies and can be industrialized to meet the demand for clinical treatment. Nevertheless, the rejection potential of transplantation of allogeneic MSCs may lead to the loss of MSCs' ability to relieve IBD [58, 126]. Therefore, which type of MSCs to choose and whether to use autologous or allogeneic MSCs should depend on clinical condition and actual demand.

### 8.2. Technical Development of Large-Scale Expansion of MSCs In Vitro

Technologies and machines for tissue extraction, separation, and purification and in vitro amplification of MSCs were getting more and more mature to meet the growing clinical needs. The water-jet assisted liposuction was employed to adipose tissue collection and the clean room environment was applied to culture ADMSCs in vitro to preserve a high cell viability [127]. Moreover, many organizations have been working on the development of innovative cellular medicines, and their products have achieved consistent and long-lasting clinical outcomes in a variety of difficult-to-treat diseases. A team in Australia relieved the progression of disc degeneration in an ovine model via mesenchymal precursor cells (MPCs), which is a kind of earliest uncommitted clonogenic populations of bone marrow stromal cells. They prepared the MPCs which were supported from Mesoblast Ltd., a world leader company of cellular medicines [128]. The company owns proprietary good manufacturing practice (GMP) procedures and product release criteria which are approved by the TGA, and the MPCs they supplied can be free from donor recipient matching or recipient immunosuppression. A research they supported on the safety and effectiveness of clinical treatment of mesenchymal stem cells has been practiced into a controlled double-blind randomised trial [129]. In order to provide sufficient quantities for experimental and commercial use, Cynata Therapeutics Ltd. have adopted Cymerus technology to make pluripotent stem cells (iPSCs) differentiate into precursor cells and then into mesenchymal stem cells (MCA-MSCs) [130]. With the development of modern technology and research, the opportunities for mesenchymal stem cell medicine for clinical application of IBD will increase greatly in the future.

### 8.3. When, How, and in What Doses of MSCs Administration

Over the last decade or so, there were no uniform standards about the time of administration, mode of administration, and dose of administration for MSCs in IBD. As is described above, certain inflammatory factors may promote the function of MSCs to alleviate IBD in the inflammatory microenvironment. When to administer MSCs should be considered since IBD is an inflammatory burst and a reversal of alternating chronic diseases [131]. The clinical efficacy of different MSCs administration methods was not very similar, including intravenous infusion and local injection. Intravenous administration was suitable for systemic UC and CD, whereas local injection was appropriate for perianal fistula. Data have shown that patients with ulcerative colitis achieved the remission of the clinical and morphological indices of inflammatory activity via a single dose of (1.5-2) x 10^8^ of BMMSCs intravenously [112]. Twenty-four patients with severe anal fistula received local injections of ADMSCs and the results showed that half of the patients were rid of the reoperation and some of them gained that abscesses and the fistulas were cured [132]. In the preclinical phase, different doses of MSCs have different therapeutic effects. However, studies have found that single infusion of BMMSCs which propagated with human platelet lysate-supplemented media was safe and feasible at the doses of up to 10 million cells/kg [120]. For anal fistula, the researchers increased the dose to 40 million after 8 weeks of local injection of 20 million MSCs failed, and finally the fistula was sealed. But it is not convincing enough because of the lack of data [114]. An attempt was made to inject MSCs into the fistula continuously every four weeks, with a total 4 doses, which also suppressed rectal inflammation [133].

To sum up, the course of IBD disease is long and reiterant, so when, how, and in what doses of MSCs administration used to treat enteritis should be formulated according to the patient's own disease indications.

### 8.4. Potential Side Effects and Challenges

To date, no significant adverse event has been reported in clinical studies of MSCs in the treatment of IBD. Intravenous MSCs may cause transient fever, as well as other side effects, such as insomnia and increased diarrhea, but all can be self-cured [134]. But the most concerning was whether MSCs carry the virus, which inhibits the effectiveness of treating enteritis and even causes infectious diseases [135]. There are also many challenges to be solved in the treatment of MSCs of enteritis such as how to improve the survival rate of MSCs in* in vivo* inflammatory environment and how to ensure safety and nonneoplasm after long-term administration of MSCs [11]. Meanwhile, the therapeutic mechanism of MSCs in human body needs to be elaborated in order to improve treatment regimens. Although clinical data are not complete at present, effective results of MSCs therapy have been made in phase III clinical trials in IBD.

## 9. Conclusion and Discussion

The therapy of MSCs has been gradually matured in the animal model stage for the treatment of enteritis. MSCs whether from different tissue sources or engineered with genes or chemical modifications to improve immune suppression and cell survival have their own unique therapeutic effects. This was achieved by MSCs through cell to cell and paracrine. MSCs homed to the intestinal mucosa and differentiated into epithelium and other cells. Meanwhile, paracrine active substances inhibited the proliferation and promoted the apoptosis of Th1 and Th17 cells and other immune cells so as to inhibit excessive immunity. Exosomes, a kind of representative of MSCs paracrine, have also been detected to be capable of replacing MSCs in the treatment of enteritis in recent years [96]. Moreover, exosomes are easy to store, transport, industrialize, and avoid the risk of immune rejection [136]. It has been reported that the function of exosomes to protect the kidney from ischemia-reperfusion induced injury would be lost when RNA in exosomes was removed via ribonuclease [137]. Our previous studies have demonstrated that exosomes derived from HUCMSCs promoted wnt/*β*-catenin expression and thereby boosted wound repair, and this function would be enhanced by exosomes derived from 3,3-diindolylmethane (DIM) pretreated HUCMSCs [138, 139]. Those results indicated that the miRNA, mRNA, and bioactive proteins carried by exosomes were critical for tissue injury repair. As is indicated above, exosomes themselves have the function of repairing intestinal mucosal damage similar to MSCs, and exosomes secreted by engineered MSCs may also play a greater role in enteritis.

In exceptional circumstances, MSCs also have a certain therapeutic effect on colorectal cancer. MSCs were able to prolong patients survival time who suffered with metastatic tumors and complications caused by colorectal cancer [36]. Moreover, MSCs also can be regarded as gene delivery vector targeting solid tumor tissue to suppress tumor growth specifically, which is owing to abilities of targeting tumor and immune suppression [103]. MSCs can slow CAC development via inhibiting chronic inflammation, as well as playing a role of chemoprophylaxis [10]. Actually, all of the applications of MSCs in colorectal cancer need to determined according to the practical situation.

Phase III clinical trials of MSCs for the treatment of UC and CD have achieved some positive results so far [113]. While BMMSCs were used a lot in IBD, the clinical application of other tissues was also gradually increased, such as ADMSCs and UCMSCs. At the same time, some challenges need to be gradually addressed. It is urgent to improve the organizational donation channel, ensure the safe amplification of MSCs* in vitro*, and establish standards for the identification of the functions and activity of MSCs. Furthermore, existing clinical experience needs to be summarized to develop individualized administration standards of MSCs therapy for IBD, including administration time, method, dose, and frequency. Certainly, the potential side effects caused by MSCs therapy should not be ignored. If these challenges are overcome to the maximum, MSCs treatment will benefit not only IBD patients but also other chronic immune inflammation.

## Figures and Tables

**Table 1:** The therapeutic effect of different source of MSCs in experimental IBD and CAC with different engineering treatments.

	**Source**	**Engineering treatments**	**Infusion method**	**Outcome**	**Reference**
1	M-ADMSCs	macrophages	IP	↓ mortality and weight loss, inflammatory cytokines	[24]

2	H-GMSCs	Acetylsalicylic Acid		↓ disease phenotypes	[59]

3	M-BMMSCs	Aspirin	IV	↓DAI and colonic inflammation	[45]

4	H-UCB-MSCs	DNA methyltransferase		↑ therapeutic effect of MSCs	[51]

5	R-BMMSCs	G-CSF	IV	↓ DAI, MPO activity, serum TNF-a and NF-*κ*B p65	[60]

6	H-ADMSCs	HCB platelet lysate	enema	↓ colitis scores, inflamed area and inflammatory mediators.	[61]

7	M-BMMSCs	Heparin	IV	↓ mortality,weight loss, inflammation reaction and tissue injury	[62]

8	H-UCMSCs	IFN-*γ* overexpression	IV	↓ the ease of body weight,↑ colon length, ↓DAI, and↑ small intestine tissues structure	[49]

9	M-BMMSCs	Inhibition of Gal-3	IP	↑ therapeutic potential	[63]

10	M-BMMSCs	Insulin-like Growth Factor Binding Protein 7	IV and IP	↓ clinical and histopathological severity	[64]

11	H-BMMSCs	knocking down let-7a	IV	↑ therapeutic potential	[65]

12	H-BMMSCs	low doses of ultraviolet radiation and X-rays		↑ the effect of radiotherapy in CRC	[66]

13	M-BMMSCs	MicroRNA Let-7a Knockdown	IV	↓ mortality, ↓ weight loss,↓ inflammation reaction,↓ tissue lesion	[65]

14	R-ADMSCs	miR-1236 knock down	enema	↓ inflammation markers	[23]

15	Microvesicles M-BMSCs	miR-200b overexpression	IV	↑ the colon fibrosis histologically	[67]

16	M-BMMSCs	miR-21 Knockdown	IV	↑ colonic inflammation in a TGF-b1-dependent manner	[48]

17	R-BMMSCs	Over-expression CXCR4	IV	↓ both clinical and microanatomical severity of colitis	[50]

18	H-UCMSCs	Poly (I:C)	IP	↑ clinical and pathological manifestations	[68]

19	M-BMMSCs	Poly(I:C)	IP	↓ the pathologic severity	[69]

20	H-UCMSCs	poly(I:C) and LPS	IP	↓ clinical signs of disease, colon shortening and histological disease	[53]

21	R/H-BMMSCs	melatonin	IV	↑ therapeutic effect	[55]

22	M-BMMSCs	spheroid formation	Intraluminal	↓ body weight loss and ↓ DAI	[57]

23	H-BMSCs	Spheroidal formation	IP	simplify long-distance transportation and ↑ therapeutic application	[56]

24	BMMSCs	transfect with sodium iodide symporter	IV	↓ tumor growth and ↑ overall survival	[66]

25	R-BMMSCs	Tregs	IV	↓clinical and histopathologic severity	[10]

26	M-BMMSCs	Tregs cell	IV	↑ body weight and ↑ colon morphology	[70]

M, mouse; H, human; R, rat; IP, intraperitoneal injection; IV, intravenous injection; NEC, necrotising enterocolitis.

**Table 2:** The therapeutic theory of different sources of MSCs.

**BMMSCs**	**ADMSCs**	**UCBMSCs**	**UCMSCs**	**TDMSCs**	**AMSCs**	**AFSC**
↑ Treg cells	↑ Treg cells	↑ Treg cells	↑ Treg cells	↓CD3+T cells	↓Macrophage	↑ Treg cells
↑ Th2 cells	↑ Th2 cells	↑ Th2 cells	↑ Th2 cells	(↑apoptosis	(M1→M2)	↑ Th2 cells
↓ Th1 cells	↓ Th1 cells	↓ Th1 cells	↓ Th1 cells	↓proliferation)	↓ Neutrophils	↓ Th1 cells
↓ Th17 cells	↓ Th17 cells	↓ Th17 cells	↓ Th17 cells			↓ Th17 cells
↓Macrophage (M1→M2)	↓Macrophage	regulate tight junction proteins	↓CD3+T cells			↑ COXs
↓ Neutrophils	(M1→M2)	↓ monocyte	(↑apoptosis			
↑ MDSCs	↓oxidative stress	↓CD3+T cells activation	↓proliferation)			
↓CD3+T cells	↓cell senescence		↓ Neutrophils			
(↑apoptosis ↓proliferation)	↑wound repair		↑ CD5+ B cells			
↓oxidative stress			↑ CD5+ Bregs			
↓cell senescence						
↓mucosal permeability						
↑neuroprotective						

**MDSCs: **myeloid derived suppressor cells.

**Table 3:** CLINICAL data of representative MSCs therapy for IBD from *Pubmed* in the past decade.

	Sources	Disease	Therapeutic schedule	Phase	Number	Evaluation	Outcome	Reference
1	Allogeneic- BMMSCs	UC	a single dose of (1.5-2) x 10^8^ intravenously	II	44	24 months	34 (72.7%) of patients achieved the clinical and morphological indices of inflammatory activity↓.	[112]

2	Allogeneic- ADMSCs	Perianal fistulizing CD	a single dose of 1.2 x 10^8^ intralesionally	III	212	24 weeks	50% of patients treated with ADMSCs achieved combined remission.	[113]

3	Allogeneic- ADMSCs	Perianal fistulizing CD	a single dose intralesionally If 2 x 10^7^ failed, 4 x 10^7^ subsequently	II	24	24 weeks	69.2% of patients achieved the number of draining fistulas↓ 56.3% of patients achieved complete fistula closure of the treated. 30% of patients achieved complete closure of all existing fistula tracts.	[114]

4	Allogeneic- BMMSCs	Perianal fistulizing CD	a single dose of 1 × 10^7^, 3 × 10^7^, or 9 × 10^7^ intralesionally	II	21	6, 12, and 24 weeks	no severe adverse events. 3 × 10^7^ MSCs appeared to promote healing of perianal fistulas	[115]

5	Allogeneic-MSCs	Luminal CD	a single dose of 2 × 10^6^ /kg weekly for 4 weeks intravenously	II	16	42 days	15 patients CDAI score ↓ from 370 to 203 at day 42	[116]

6	Autologous-BMMSCs	Luminal CD	two doses of 1-2×10^6^ intravenously, 7 days apart	I	10	0, 6 weeks	3 patients CDAI score ↓ at 6 weeks	[117]

7	Autologous-ADMSCs	Perianal fistulizing CD	local injection,2×10^7^	I	12	24 weeks	9 of 12 patients had complete clinical healing by 3 months, 10 of 12 patients (83.3%) had complete clinical healing at 6 months	[118]

8	Autologous-ADMSCs	Perianal fistulizing CD	group 1: 1 × 10^7^ cells/ml. After 4 weeks, if safe, group 2: 2 × 10^7^ cells/ml. After 4 weeks, if safe, group 3: 4 × 10^7^ cells/ml	I	>9	8 weeks	2 patients in group 2 showed complete healing and 3 patients in group 3 showed complete healing at week 8.	[119]

9	Autologous-BMMSCs	CD	a single dose of 2 × 10^7^, 5 × 10^7^, or 10 × 10^7^ cells/kg intravenously	I	12	2 weeks	Single infusion of BMMSCs propagated ex vivo using human platelet lysate-supplemented media was safe and feasible at the doses of up to 10 million cells/kg	[120]

10	Autologous-ADMSCs	Perianal fistulizing CD	3 × 10^7^ cells per centimeter length	II	43	8 weeks	27/33 patients (82%) had complete fistula healing. Of 27 patients, 23 patients (88%) sustained complete closure	[121]

## References

[1] Desai D., Desai N. (2014). Colorectal cancer surveillance in inflammatory bowel disease: A critical analysis. *World Journal of Gastrointestinal Endoscopy*.

[2] Gao R., Gao Z., Huang L., Qin H. (2017). Gut microbiota and colorectal cancer. *European Journal of Clinical Microbiology & Infectious Diseases Official Publication of the European Society of Clinical Microbiology*.

[3] De Francesco F., Romano M., Zarantonello L. (2016). The role of adipose stem cells in inflammatory bowel disease: From biology to novel therapeutic strategies. *Cancer Biology & Therapy*.

[4] Nicholls R. J. (2002). Ulcerative colitis - Surgical indications and treatment. *Alimentary Pharmacology and Therapeutics*.

[5] Rubin D. T., LoSavio A., Yadron N., Huo D., Hanauer S. B. (2006). Aminosalicylate therapy in the prevention of dysplasia and colorectal cancer in ulcerative colitis. *Clinical Gastroenterology and Hepatology*.

[6] Hanauer S. B., Feagan B. G., Lichtenstein G. R. (2002). Maintenance infliximab for Crohn's disease: the ACCENT I randomised trial. *The Lancet*.

[7] Dave M., Mehta K., Luther J., Baruah A., Dietz A. B., Faubion W. A. (2015). Mesenchymal stem cell therapy for inflammatory bowel disease: A systematic review and meta-analysis. *Inflammatory Bowel Diseases*.

[8] Di Franco S., Todaro M., Dieli F., Stassi G. (2013). Colorectal cancer defeating? Challenge accepted!. *Molecular Aspects of Medicine*.

[9] Ricart E., Jauregui-Amezaga A., Ordás I., Pinó S., Ramírez A. M., Panés J. (2013). Cell therapies for IBD: what works?. *Current Drug Targets*.

[10] Tang R. J., Shen S. N., Zhao X. Y. (2015). Mesenchymal stem cells-regulated Treg cells suppress colitis-associated colorectal cancer. *Stem Cell Research & Therapy*.

[11] Chinnadurai R., Ng S., Velu V., Galipeau J. (2015). Challenges in animal modelling of mesenchymal stromal cell therapy for inflammatory bowel disease. *World Journal of Gastroenterology*.

[12] Okamoto R., Watanabe M. (2016). Investigating cell therapy for inflammatory bowel disease. *Expert Opinion on Biological Therapy*.

[13] Galipeau J. (2018). Mesenchymal Stromal Cells. *Hematology*.

[14] Caprioli F., Pallone F., Monteleone G. (2008). Th17 immune response in IBD: A new pathogenic mechanism. *Journal of Crohn's and Colitis*.

[15] De Souza H. S. P., Fiocchi C., Iliopoulos D. (2017). The IBD interactome: An integrated view of aetiology, pathogenesis and therapy. *Nature Reviews Gastroenterology & Hepatology*.

[16] Neofytou E., Deuse T., Beygui R. E., Schrepfer S. (2015). Mesenchymal stromal cell therapy: Different sources exhibit different immunobiological properties. *Transplantation*.

[17] Sun T., Gao G. Z., Li R. F. (2015). Bone marrow-derived mesenchymal stem cell transplantation ameliorates oxidative stress and restores intestinal mucosal permeability in chemically induced colitis in mice. *American Journal of Translational Research*.

[18] Robinson A. M., Rahman A. A., Miller S., Stavely R., Sakkal S., Nurgali K. (2017). The neuroprotective effects of human bone marrow mesenchymal stem cells are dose-dependent in TNBS colitis. *Stem Cell Research & Therapy*.

[19] Lee H. J., Oh S.-H., Jang H. W. (2016). Long-term effects of bone marrow-derived mesenchymal stem cells in dextran sulfate sodium-induced murine chronic colitis. *Gut and Liver*.

[20] Ciccocioppo R., Cangemi G. C., Kruzliak P., Corazza G. R. (2016). Cellular therapies: the potential to regenerate and restore tolerance in immune-mediated intestinal diseases. *Stem Cells*.

[21] Bassi G., Pacelli L., Carusone R., Zanoncello J., Krampera M. (2012). Adipose-derived stromal cells (ASCs). *Transfusion and Apheresis Science*.

[22] Nakao N., Nakayama T., Yahata T. (2010). Adipose tissue-derived mesenchymal stem cells facilitate hematopoiesis in vitro and in vivo: advantages over bone marrow-derived mesenchymal stem cells. *The American Journal of Pathology*.

[23] Zhang Y., Jin Y., Lin Y. (2015). Adipose-Derived Mesenchymal Stem Cells Ameliorate Ulcerative Colitis Through MIR-1236 Negatively Regulating the Expression of Retinoid-Related Orphan Receptor Gamma. *DNA and Cell Biology*.

[24] Anderson P., Souza-Moreira L., Morell M. (2013). Adipose-derived mesenchymal stromal cells induce immunomodulatory macrophages which protect from experimental colitis and sepsis. *Gut*.

[25] Lopez-Santalla M., Mancheño-Corvo P., Escolano A. (2017). Biodistribution and efficacy of human adipose-derived mesenchymal stem cells following intranodal administration in experimental colitis. *Frontiers in Immunology*.

[26] Xie M., Qin H., Luo Q., He X., Lan P., Lian L. (2016). Comparison of adipose-derived and bone marrow mesenchymal stromal cells in a murine model of Crohn's disease. *Digestive Diseases & Sciences*.

[27] Stavely R., Robinson A. M., Miller S., Boyd R., Sakkal S., Nurgali K. (2015). Human adult stem cells derived from adipose tissue and bone marrow attenuate enteric neuropathy in the guinea-pig model of acute colitis. *Stem Cell Research & Therapy*.

[28] Zhang B., Wu X., Zhang X. (2015). Human umbilical cord mesenchymal stem cell exosomes enhance angiogenesis through the Wnt4/*β*-catenin pathway. *Stem Cells Translational Medicine*.

[29] Wang M., Cai J., Huang F. (2015). Pre-treatment of human umbilical cord-derived mesenchymal stem cells with interleukin-6 abolishes their growth-promoting effect on gastric cancer cells. *International Journal of Molecular Medicine*.

[30] Cao H., Qian H., Xu W. (2010). Mesenchymal stem cells derived from human umbilical cord ameliorate ischemia/reperfusion-induced acute renal failure in rats. *Biotechnology Letters*.

[31] Kim H.-S., Shin T.-H., Lee B.-C. (2013). Human umbilical cord blood mesenchymal stem cells reduce colitis in mice by activating NOD2 signaling to COX2. *Gastroenterology*.

[32] Baksh D., Yao R., Tuan R. S. (2007). Comparison of proliferative and multilineage differentiation potential of human mesenchymal stem cells derived from umbilical cord and bone marrow. *Stem Cells*.

[33] Mao F., Wu Y., Tang X. (2017). Exosomes derived from human umbilical cord mesenchymal stem cells relieve inflammatory bowel disease in mice. *BioMed Research International*.

[34] Lv Y., Xu X., Zhang B. (2014). Endometrial regenerative cells as a novel cell therapy attenuate experimental colitis in mice. *Journal of Translational Medicine*.

[35] Zani A., Cananzi M., Fascetti-Leon F. (2014). Amniotic fluid stem cells improve survival and enhance repair of damaged intestine in necrotising enterocolitis via a COX-2 dependent mechanism. *Gut*.

[36] Zhang D., Zheng L., Shi H. (2014). Suppression of peritoneal tumorigenesis by placenta-derived mesenchymal stem cells expressing endostatin on colorectal cancer. *International Journal of Medical Sciences*.

[37] Miyamoto S., Ohnishi S., Onishi R. (2017). Therapeutic effects of human amnion-derived mesenchymal stem cell transplantation and conditioned medium enema in rats with trinitrobenzene sulfonic acid-induced colitis. *American Journal of Translational Research*.

[38] Legaki E., Roubelakis M. G., Theodoropoulos G. E. (2016). Therapeutic potential of secreted molecules derived from human amniotic fluid mesenchymal stem/stroma cells in a mice model of colitis. *Stem Cell Reviews and Reports*.

[39] Xu X., Chen C., Akiyama K. (2013). Gingivae contain neural-crest- and mesoderm-derived mesenchymal stem cells. *Journal of Dental Research*.

[40] Yu Y., Song E. M., Lee K. E. (2017). Therapeutic potential of tonsil-derived mesenchymal stem cells in dextran sulfate sodium-induced experimental murine colitis. *PLoS ONE*.

[41] Lee J. M., Jung J., Lee H.-J. (2012). Comparison of immunomodulatory effects of placenta mesenchymal stem cells with bone marrow and adipose mesenchymal stem cells. *International Immunopharmacology*.

[42] Phinney D. G. (2012). Functional heterogeneity of mesenchymal stem cells: implications for cell therapy. *Journal of Cellular Biochemistry*.

[43] Ankrum J., Karp J. M. (2010). Mesenchymal stem cell therapy: two steps forward, one step back. *Trends in Molecular Medicine*.

[44] Fox I. J., Daley G. Q., Goldman S. A., Huard J., Kamp T. J., Trucco M. (2014). Stem cell therapy. Use of differentiated pluripotent stem cells as replacement therapy for treating disease. *Science*.

[45] Tang J., Xiong J., Wu T. (2014). Aspirin Treatment Improved Mesenchymal Stem Cell Immunomodulatory Properties via the 15d-PGJ2/PPARÎ^3^/TGF-Î^2^1 Pathway. *Stem Cells & Development*.

[46] Hoffman A. M., Dow S. W. (2016). Concise review: stem cell trials using companion animal disease models. *Stem Cells*.

[47] Neudecker V., Haneklaus M., Jensen O. (2017). Myeloid-derived miR-223 regulates intestinal inflammation via repression of the NLRP3 inflammasome. *The Journal of Experimental Medicine*.

[48] Wu T., Liu Y., Fan Z. (2015). miR-21 modulates the immunoregulatory function of bone marrow mesenchymal stem cells through the PTEN/Akt/TGF-*β*1 pathway. *Stem Cells*.

[49] Chen Y., Song Y., Miao H. (2015). Gene delivery with IFN-*γ*-expression plasmids enhances the therapeutic effects of MSCs on DSS-induced mouse colitis. *Inflammation Research*.

[50] Liu X., Zuo D., Fan H. (2014). Over-expression of CXCR4 on mesenchymal stem cells protect against experimental colitis via immunomodulatory functions in impaired tissue. *Journal of Molecular Histology*.

[51] Lee S., Kim H.-S., Roh K.-H. (2015). DNA methyltransferase inhibition accelerates the immunomodulation and migration of human mesenchymal stem cells. *Scientific Reports*.

[52] Yu K., Lee J. Y., Kim H. (2014). A p38 MAPK-mediated alteration of COX-2/PGE_2_ regulates immunomodulatory properties in human mesenchymal stem cell aging. *PLoS ONE*.

[53] Fuenzalida P., Kurte M., Fernández-O'ryan C. (2016). Toll-like receptor 3 pre-conditioning increases the therapeutic efficacy of umbilical cord mesenchymal stromal cells in a dextran sulfate sodium–induced colitis model. *Cytotherapy*.

[54] Irhimeh M. R., Cooney J. (2016). Management of inflammatory bowel disease using stem cell therapy. *Current Stem Cell Research & Therapy*.

[55] Shuai Y., Liao L., Su X. (2016). Melatonin treatment improves mesenchymal stem cells therapy by preserving stemness during long-term in vitro expansion. *Theranostics*.

[56] Jiang B., Yan L., Miao Z., Li E., Wong K. H., Xu R.-H. (2017). Spheroidal formation preserves human stem cells for prolonged time under ambient conditions for facile storage and transportation. *Biomaterials*.

[57] Molendijk I., Barnhoorn M. C., de Jonge-Muller E. S. M. (2016). Intraluminal Injection of Mesenchymal Stromal Cells in Spheroids Attenuates Experimental Colitis. *Journal of Crohn's and Colitis*.

[58] Moll G., Rasmusson-Duprez I., von Bahr L. (2012). Are therapeutic human mesenchymal stromal cells compatible with human blood?. *Stem Cells*.

[59] Liu Y., Chen C., Liu S. (2015). Acetylsalicylic acid treatment improves differentiation and immunomodulation of SHED. *Journal of Dental Research*.

[60] Tang Y., Chen Y., Wang X., Song G., Li Y., Shi L. (2015). Combinatorial Intervention with Mesenchymal Stem Cells and Granulocyte Colony-Stimulating Factor in a Rat Model of Ulcerative Colitis. *Digestive Diseases and Sciences*.

[61] Forte D., Ciciarello M., Valerii M. C. (2015). Human cord blood-derived platelet lysate enhances the therapeutic activity of adipose-derived mesenchymal stromal cells isolated from Crohn's disease patients in a mouse model of colitis. *Stem Cell Research & Therapy*.

[62] Liao L., Shi B., Chang H. (2017). Heparin improves BMSC cell therapy: Anticoagulant treatment by heparin improves the safety and therapeutic effect of bone marrow-derived mesenchymal stem cell cytotherapy. *Theranostics*.

[63] Markovic B. S., Nikolic A., Gazdic M. (2016). Pharmacological inhibition of Gal-3 in mesenchymal stem cells enhances their capacity to promote alternative activation of macrophages in dextran sulphate sodium-induced colitis. *Stem Cells International*.

[64] Robinson A., Miller S., Sakkal S. (2014). Mesenchymal stem cells and conditioned medium avert inflammation-induced enteric neuropathy. *Neurogastroenterology & Motility*.

[65] Yu Y., Liao L., Shao B. (2017). Knockdown of MicroRNA Let-7a Improves the Functionality of Bone Marrow-Derived Mesenchymal Stem Cells in Immunotherapy. *Molecular Therapy*.

[66] Knoop K., Schwenk N., Schmohl K. (2015). Mesenchymal stem cell-mediated, tumor stroma-targeted radioiodine therapy of metastatic colon cancer using the sodium iodide symporter as theranostic gene. *Journal of Nuclear Medicine*.

[67] Yang J., Zhou C. H., Zhu R. (2017). Microvesicles shuttled mir0b attenuate experimental colitis associated intestinal fibrosis by inhibiting the development of EMT. *Journal of Gastroenterology & Hepatology*.

[68] Ryu D.-B., Lim J.-Y., Lee S.-E., Park G., Min C.-K. (2016). Induction of indoleamine 2,3-dioxygenase by pre-treatment with poly(I:C) may enhance the efficacy of MSC treatment in DSS-induced colitis. *Immune Network*.

[69] Qiu Y., Guo J., Mao R. (2017). TLR3 preconditioning enhances the therapeutic efficacy of umbilical cord mesenchymal stem cells in TNBS-induced colitis via the TLR3-Jagged-1-Notch-1 pathway. *Mucosal Immunology*.

[70] Yu Y., Zhao T., Yang D. (2017). Cotransfer of regulatory T cells improve the therapeutic effectiveness of mesenchymal stem cells in treating a colitis mouse model. *Journal of Experimental Animal Science*.

[71] Cho D. I., Mi R. K., Jeong H. (2014). Mesenchymal stem cells reciprocally regulate the M1|[sol]|M2 balance in mouse bone marrow-derived macrophages. *Experimental Molecular Medicine*.

[72] Holan V., Hermankova B., Bohacova P. (2016). Distinct Immunoregulatory Mechanisms in Mesenchymal Stem Cells: Role of the Cytokine Environment. *Stem Cell Reviews and Reports*.

[73] Wang M., Liang C., Hu H. (2016). Intraperitoneal injection (IP), Intravenous injection (IV) or anal injection (AI)? Best way for mesenchymal stem cells transplantation for colitis. *Scientific Reports*.

[74] González M. A., Gonzalez-Rey E., Rico L., Büscher D., Delgado M. (2009). Adipose-derived mesenchymal stem cells alleviate experimental colitis by inhibiting inflammatory and autoimmune responses. *Gastroenterology*.

[75] Ko I. K., Kim B.-G., Awadallah A. (2010). Targeting improves MSC treatment of inflammatory bowel disease. *Molecular Therapy*.

[76] Zheng K., Wu W., Yang S. (2016). Treatment of radiation-induced acute intestinal injury with bone marrow-derived mesenchymal stem cells. *Experimental and Therapeutic Medicine*.

[77] Yemm A., Adams D., Kalia N. (2015). Targeting the delivery of systemically administered haematopoietic stem/progenitor cells to the inflamed colon using hydrogen peroxide and platelet microparticle pre-treatment strategies. *Stem Cell Research*.

[78] Ren G., Zhang L., Zhao X. (2008). Mesenchymal stem cell-mediated immunosuppression occurs via concerted action of chemokines and nitric oxide. *Cell Stem Cell*.

[79] Chen Q.-Q., Yan L., Wang C.-Z. (2013). Mesenchymal stem cells alleviate TNBS-induced colitis by modulating inflammatory and autoimmune responses. *World Journal of Gastroenterology*.

[80] de Miguel M. P., Fuentes-Julián S., Blázquez-Martínez A. (2012). Immunosuppressive properties of mesenchymal stem cells: advances and applications. *Current Molecular Medicine*.

[81] Chao K., Zhang S., Qiu Y. (2016). Human umbilical cord-derived mesenchymal stem cells protect against experimental colitis via CD5+ B regulatory cells. *Stem Cell Research & Therapy*.

[82] Sala E., Genua M., Petti L. (2015). Mesenchymal Stem Cells Reduce Colitis in Mice via Release of TSG6, Independently of Their Localization to the Intestine. *Gastroenterology*.

[83] Wang L.-T., Ting C.-H., Yen M.-L. (2016). Human mesenchymal stem cells (MSCs) for treatment towards immune- and inflammation-mediated diseases: review of current clinical trials. *Journal of Biomedical Science*.

[84] Wang Y., Chen X., Cao W., Shi Y. (2014). Plasticity of mesenchymal stem cells in immunomodulation: pathological and therapeutic implications. *Nature Immunology*.

[85] Zuo D., Tang Q., Fan H. (2015). Modulation of nuclear factor-*κ*B-mediated pro-inflammatory response is associated with exogenous administration of bone marrow-derived mesenchymal stem cells for treatment of experimental colitis. *Molecular Medicine Reports*.

[86] Fan H., Zhao G., Liu L. (2012). Pre-treatment with IL-1*β* enhances the efficacy of MSC transplantation in DSS-induced colitis. *Cellular & Molecular Immunology*.

[87] Yu Y., Shao B., Shuai Y. (2013). Role of bone marrow-derived mesenchymal stem cells in treating colitis through Fas/FasL-mediated immune regulation. *Chinese Journal of Cellular and Molecular Immunology*.

[88] Tran T.-H., Mattheolabakis G., Aldawsari H., Amiji M. (2015). Exosomes as nanocarriers for immunotherapy of cancer and inflammatory diseases. *Clinical Immunology*.

[89] Greening D. W., Gopal S. K., Xu R., Simpson R. J., Chen W. (2015). Exosomes and their roles in immune regulation and cancer. *Seminars in Cell & Developmental Biology*.

[90] Lai R. C., Yeo R. W. Y., Lim S. K. (2015). Mesenchymal stem cell exosomes. *Seminars in Cell & Developmental Biology*.

[91] Burrello J., Monticone S., Gai C., Gomez Y., Kholia S., Camussi G. (2016). Stem cell-derived extracellular vesicles and immune-modulation. *Frontiers in Cell Developmental Biology*.

[92] Lin K.-C., Yip H.-K., Shao P.-L. (2016). Combination of adipose-derived mesenchymal stem cells (ADMSC) and ADMSC-derived exosomes for protecting kidney from acute ischemia-reperfusion injury. *International Journal of Cardiology*.

[93] Arslan F., Lai R. C., Smeets M. B. (2013). Mesenchymal stem cell-derived exosomes increase ATP levels, decrease oxidative stress and activate PI3K/Akt pathway to enhance myocardial viability and prevent adverse remodeling after myocardial ischemia/reperfusion injury. *Stem Cell Research*.

[94] Mao F., Wu Y., Tang X. (2017). Human umbilical cord mesenchymal stem cells alleviate inflammatory bowel disease through the regulation of 15-LOX-1 in macrophages. *Biotechnology Letters*.

[95] Yang J., Liu X.-X., Fan H. (2015). Extracellular vesicles derived from bone marrow mesenchymal stem cells protect against experimental colitis via attenuating colon inflammation, oxidative stress and apoptosis. *PLoS ONE*.

[96] Rager T. M., Olson J. K., Zhou Y., Wang Y., Besner G. E. (2016). Exosomes secreted from bone marrow-derived mesenchymal stem cells protect the intestines from experimental necrotizing enterocolitis. *Journal of Pediatric Surgery*.

[97] Record M., Carayon K., Poirot M., Silvente-Poirot S. (2014). Exosomes as new vesicular lipid transporters involved in cell-cell communication and various pathophysiologies. *Biochimica et Biophysica Acta (BBA) - Molecular and Cell Biology of Lipids*.

[98] Collino F., Deregibus M. C., Bruno S. (2010). Microvesicles derived from adult human bone marrow and tissue specific mesenchymal stem cells shuttle selected pattern of miRNAs. *PLoS ONE*.

[99] Ti D., Hao H., Fu X., Han W. (2016). Mesenchymal stem cells-derived exosomal microRNAs contribute to wound inflammation. *Science China Life Sciences*.

[100] Yanez-Mo M., Siljander P. R., Andreu Z. (2015). Biological properties of extracellular vesicles and their physiological functions. *Journal of Extracellular Vesicles*.

[101] Jemal A., Bray F., Center M. M., Ferlay J., Ward E., Forman D. (2015). Global cancer statistics. *Ca A Cancer Journal for Clinicians*.

[102] Koliaraki V., Pallangyo C. K., Greten F. R., Kollias G. (2017). Mesenchymal Cells in Colon Cancer. *Gastroenterology*.

[103] Bajetto A., Pattarozzi A., Corsaro A. (2017). Different effects of human umbilical cord mesenchymal stem cells on glioblastoma stem cells by direct cell interaction or via released soluble factors. *Frontiers in Cellular Neuroscience*.

[104] Yang L., Zhang Y., Cheng L. (2016). Mesenchymal stem cells engineered to secrete pigment epithelium-derived factor inhibit tumor metastasis and the formation of malignant ascites in a murine colorectal peritoneal carcinomatosis model. *Human Gene Therapy*.

[105] Keung E. Z., Nelson P. J., Conrad C. (2013). Concise review: genetically engineered stem cell therapy targeting angiogenesis and tumor stroma in gastrointestinal malignancy. *Stem Cells*.

[106] Gwendal L., Paula Y L. (2016). Recent discoveries concerning the tumor - mesenchymal stem cell interactions. *Biochimica et Biophysica Acta (BBA) - Reviews on Cancer*.

[107] Nasuno M., Arimura Y., Nagaishi K. (2014). Mesenchymal stem cells cancel azoxymethane-induced tumor initiation. *Stem Cells*.

[108] Chen Z., He X., He X. (2014). Bone marrow mesenchymal stem cells ameliorate colitis-Associated tumorigenesis in mice. *Biochemical and Biophysical Research Communications*.

[109] Feng H., Zhao J.-K., Schiergens T. S. (2018). Bone marrow-derived mesenchymal stromal cells promote colorectal cancer cell death under low-dose irradiation. *British Journal of Cancer*.

[110] Hendijani F., Javanmard S. H. (2015). Dual protective and cytotoxic benefits of mesenchymal stem cell therapy in combination with chemotherapy/radiotherapy for cancer patients. *Critical Reviews in Eukaryotic Gene Expression*.

[111] Drakos P. E., Nagler A., Or R. (1993). Case of Crohn's disease in bone marrow transplantation. *American Journal of Hematology*.

[112] Kniazev O. V., Parfenov A. I., Ruchkina I. N., Lazebnik L. B., Sagynbaeva V. É. (2013). Immune response to biological therapy of inflammatory bowel diseases. *Terapevticheskii Arkhiv*.

[113] Panés J., Garcia-Olmo D., Van Assche G. (2016). Expanded allogeneic adipose-derived mesenchymal stem cells (Cx601) for complex perianal fistulas in Crohn's disease: a phase 3 randomised, double-blind controlled trial. *The Lancet*.

[114] De La Portilla F., Alba F., García-Olmo D., Herrerías J. M., González F. X., Galindo A. (2013). Expanded allogeneic adipose-derived stem cells (eASCs) for the treatment of complex perianal fistula in Crohn's disease: results from a multicenter phase I/IIa clinical trial. *International Journal of Colorectal Disease*.

[115] Molendijk I., Bonsing B. A., Roelofs H. (2015). Allogeneic bone marrow–derived mesenchymal stromal cells promote healing of refractory perianal fistulas in patients with Crohn's disease. *Gastroenterology*.

[116] Forbes G. M., Sturm M. J., Leong R. W. (2014). A phase 2 study of allogeneic mesenchymal stromal cells for luminal crohn's disease refractory to biologic therapy. *Clinical Gastroenterology and Hepatology*.

[117] Duijvestein M., Vos A. C. W., Roelofs H. (2010). Autologous bone marrow-derived mesenchymal stromal cell treatment for refractory luminal Crohn's disease: results of a phase I study. *Gut*.

[118] Dietz A. B., Dozois E. J., Fletcher J. G. (2017). Autologous mesenchymal stem cells, applied in a bioabsorbable matrix, for treatment of perianal fistulas in patients with crohn's disease. *Gastroenterology*.

[119] Cho Y. B., Lee W. Y., Park K. J., Kim M., Yoo H.-W., Yu C. S. (2013). Autologous adipose tissue-derived stem cells for the treatment of Crohn's fistula. A phase I clinical study. *Cell Transplantation*.

[120] Dhere T., Copland I., Garcia M. (2016). The safety of autologous and metabolically fit bone marrow mesenchymal stromal cells in medically refractory Crohn's disease – a phase 1 trial with three doses. *Alimentary Pharmacology & Therapeutics*.

[121] Lee W. Y., Park K. J., Cho Y. B. (2013). Autologous adipose tissue-derived stem cells treatment demonstrated favorable and sustainable therapeutic effect for crohn's fistula. *Stem Cells*.

[122] Ciccocioppo R., Bernardo M. E., Sgarella A. (2011). Autologous bone marrow-derived mesenchymal stromal cells in the treatment of fistulising Crohn's disease. *Gut*.

[123] Liang J., Zhang H., Wang D. (2012). Allogeneic mesenchymal stem cell transplantation in seven patients with refractory inflammatory bowel disease. *Gut*.

[124] Bernardo M. E., Fibbe W. E. (2015). Mesenchymal stromal cells and hematopoietic stem cell transplantation. *Immunology Letters*.

[125] Scharl M. (2014). Treatment of chronic inflammatory bowel diseases: What is next?. *Praxis*.

[126] Hoogduijn M. J., Roemeling-Van Rhijn M., Korevaar S. S., Engela A. U., Weimar W., Baan C. C. (2011). Immunological aspects of allogeneic and autologous mesenchymal stem cell therapies. *Human Gene Therapy*.

[127] Purpura V., Bondioli E., Melandri D., Parodi P. C., Valenti L., Riccio M. (2016). The collection of adipose derived stem cells using waterjet assisted lipoplasty for their use in plastic and reconstructive surgery: a preliminary study. *Frontiers in Cell Developmental Biology*.

[128] Oehme D., Ghosh P., Goldschlager T. (2016). Reconstitution of degenerated ovine lumbar discs by STRO-3–positive allogeneic mesenchymal precursor cells combined with pentosan polysulfate. *Journal of Neurosurgery: Spine*.

[129] Wang Y., Shimmin A., Ghosh P. (2017). Safety, tolerability, clinical, and joint structural outcomes of a single intra-articular injection of allogeneic mesenchymal precursor cells in patients following anterior cruciate ligament reconstruction: A controlled double-blind randomised trial. *Arthritis Research & Therapy*.

[130] Royce S. G., Rele S., Broughton B. R. S., Kelly K., Samuel C. S. (2017). Intranasal administration of mesenchymoangioblast-derived mesenchymal stem cells abrogates airway fibrosis and airway hyperresponsiveness associated with chronic allergic airways disease. *The FASEB Journal*.

[131] Wagner B., Henschler R. (2013). Fate of Intravenously Injected Mesenchymal Stem Cells and Significance for Clinical Application. *Advances in Biochemical Engineering-Biotechnology*.

[132] García-Olmo D., Herreros D., De-La-Quintana P. (2010). Adipose-derived stem cells in Crohn's rectovaginal fistula. *Case Reports in Medicine*.

[133] Ahrendt M., Hammerschmidt S. I., Pabst O., Pabst R., Bode U. (2008). Stromal cells confer lymph node-specific properties by shaping a unique microenvironment influencing local immune responses. *The Journal of Immunology*.

[134] Martínez-Montiel M. D. P., Gómez-Gómez G. J., Flores A. I. (2014). Therapy with stem cells in inflammatory bowel disease. *World Journal of Gastroenterology*.

[135] Bhatt A. S., Freeman S., Herrera A. (2013). Sequence-based discovery of Bradyrhizobium enterica in cord colitis syndrome. *Biology of Blood & Marrow Transplantation*.

[136] de Girolamo L., Lucarelli E., Alessandri G. (2013). Mesenchymal stem/stromal cells: a new ‘cells as drugs’ paradigm. Efficacy and critical aspects in cell therapy. *Current Pharmaceutical Design*.

[137] Gatti S., Bruno S., Deregibus M. C. (2011). Microvesicles derived from human adult mesenchymal stem cells protect against ischaemia-reperfusion-induced acute and chronic kidney injury. *Nephrology Dialysis Transplantation *.

[138] Zhang B., Wang M., Gong A. (2015). HucMSc-exosome mediated-Wnt4 signaling is required for cutaneous wound healing. *Stem Cells*.

[139] Shi H., Xu X., Zhang B. (2017). 3,3′-Diindolylmethane stimulates exosomal Wnt11 autocrine signaling in human umbilical cord mesenchymal stem cells to enhance wound healing. *Theranostics*.

